# Current Immunotherapeutic Strategies to Enhance Oncolytic Virotherapy

**DOI:** 10.3389/fonc.2017.00114

**Published:** 2017-06-06

**Authors:** Daniel E. Meyers, Amanda A. Wang, Chandini M. Thirukkumaran, Don G. Morris

**Affiliations:** ^1^Department of Oncology, University of Calgary, Calgary, AB, Canada; ^2^Tom Baker Cancer Centre, Calgary, AB, Canada; ^3^Cumming School of Medicine, Calgary, AB, Canada

**Keywords:** oncolytic virus, immune therapy, GM-CSF, prime boost, cyclophosphamide, immune checkpoint, sunitinib

## Abstract

Oncolytic viruses (OV) represent a promising strategy to augment the spectrum of cancer therapeutics. For efficacy, they rely on two general mechanisms: tumor-specific infection/cell-killing, followed by subsequent activation of the host’s adaptive immune response. Numerous OV genera have been utilized in clinical trials, ultimately culminating in the 2015 Food and Drug Administration approval of a genetically engineered herpes virus, Talminogene laherparepvec (T-VEC). It is generally accepted that OV as monotherapy have only modest clinical efficacy. However, due to their ability to elicit specific antitumor immune responses, they are prime candidates to be paired with other immune-modulating strategies in order to optimize therapeutic efficacy. Synergistic strategies to enhance the efficacy of OV include augmenting the host antitumor response through the insertion of therapeutic transgenes such as GM-CSF, utilization of the prime-boost strategy, and combining OV with immune-modulatory drugs such as cyclophosphamide, sunitinib, and immune checkpoint inhibitors. This review provides an overview of these immune-based strategies to improve the clinical efficacy of oncolytic virotherapy.

## Introduction

Despite the introduction of molecular interrogation and personalized medicine strategies for both the diagnosis and treatment of cancer over the past decade, the burden of this disease is still large. In 2016, an estimated 600,000 individuals died from cancer in the USA alone (1). Thus, while there is more efficacy in cancer treatment than ever before, there is still a significant potential for improvement.

Until recently, the myriad of genetic and epigenetic alterations that exist among cancer cells provided a seemingly insurmountable therapeutic challenge. How could one specific drug target all the machinery that the cancer cell uses to grow? Additionally, tumor heterogeneity and resistance mechanisms allow growth of cancer cells under the selective pressures of both the tumor microenvironment and attempted treatments (2). Thus, the answer to these treatment barriers may be in the ability to harness the potential of an equally diverse entity—the human immune system. One unique class of cancer therapeutics that utilizes the immune system is oncolytic viruses (OV).

The recognition that viral infection could play a role in the treatment of cancer first came to light over one hundred years ago (3). Only recently, though, has there been an increasing interest in the field, culminating in the US Food and Drug Administration (FDA) approval of a modified herpes simplex virus (HSV) for use in metastatic melanoma (4). There are numerous other clinical trials of OV currently ongoing (Table 1).

**Table 1:** Selected ongoing clinical trials using oncolytic viruses.

Virus	Name	Mods/effect	Tumor	Phase	Route	Combination	Trial ID
Adenovirus	DNX-2401	*Enhance viral tumor entry*: Δ24-RGD insertion	Glioma, gliosarcoma	I	IT	IFN-γ	NCT02197169
				II	IT	Pembrolizumab	NCT02798406
			Glioma	I	IT	Temozolomide	NCT01956734
	VCN-01	*Enhance intratumoral distribution*: PH20 hyaluronidase insertion	Pancreas	I	IT	Gemcitabine + Abraxane	NCT02045589
			Solid tumors	I	IV	Gemcitabine + Abraxane	NCT02045602
	Colo-Ad1	*Increase tumor specificity*: Chimeric Ad11/3 group B	Ovarian	I/II	IP	–	NCT02028117
			Solid tumors	I	IV	Nivolumab	NCT02636036
				I/II	IV	–	NCT02028442
	AdV-tk	*Increased tumor sensitivity to drug*: TK insertion	MPE	I	IPl	–	NCT01997190
			Pediatric (brain)	I	IT	RT + Valcyclovir	NCT00634231
			Pancreas	I/II	IT	Gemcitabine + RT + mFOLFIRINOX	NCT02446093
			Prostate	II/III	IT	Valcyclovir	NCT02768363
				III	IT	RT + Valcyclovir	NCT01436968
	Oncos-102	*Enhance viral tumor entry and immune activation*: Δ24-RGD-GM-CSF insertion	Melanoma	I	IT	CPA + Pembrolizumab	NCT03003676
			Mesothelioma	II	IPl	Carboplatin/Paclitaxel + CPA	NCT02879669
			Solid tumors	I	IP	Durvalumab	NCT02963831
	CG0070	*Immune activation*: GM-CSF insertion and E3 deletion	Bladder	III	Intravesicular	–	NCT02365818
Coxsackie	CVA21	None	Lung (NSLC)	I	IV	Pembrolizumab	NCT02824965
			Melanoma	I	IT	Ipilimumab	NCT02307149
					IT	Pembrolizumab	NCT02043665
			Solid tumors	I	IV	Pembrolizumab	NCT02043665
Herpes simplex	Talminogene laherparepvec	*Decreased virulence and prolong viral replication*: ICP34.5 deletion, US11 deletion, GM-CSF insertion	Breast	I/II	IT	Paclitaxel	NCT02779855
				II	IT	–	NCT02658812
			H/N	I	IT	Pembrolizumab	NCT02626000
			HCC, Liver Mets	I	IT	–	NCT02509507
			Lymphoma	II	IT	Nivolumab	NCT02978625
			Melanoma	I/II	IT	Ipilimumab	NCT01740297
				II	IT	RT	NCT02819843
						–	NCT02366195
						–	NCT02211131
						Pembrolizumab	NCT02965716
				III	IT	–	NCT02297529
						Pembrolizumab	NCT02263508
			Pediatric	I	IT	–	NCT02756845
			Sarcoma	I/II	IT	RT	NCT02453191
				II	IT	RT	NCT02923778
	HF-10	*Decreased virulence*: UL56 deletion, single partial UL52	Melanoma	II	IT	Ipilimumab	NCT02272855
			Solid tumors	I	IT	–	NCT02428036
	HSV1716	*Decreased virulence*: ICP34.5 deletion	Mesothelioma	I/II	IPl	–	NCT01721018
			Pediatric	I	IT/IV	–	NCT00931931
	G207	*Decreased virulence*: ICP34.5 deletion, UL39 disruption	Pediatric (brain)	I	IT	RT	NCT02457845
Maraba	MG1	*Tumor antigen to enhance antitumor immune activity*: MAGE-A3	Lung (NSCLC)	I/II	IM	AdMA3 Vaccine + Pembrolizumab	NCT02879760
			Solid tumors	I/II	IM	AdMA3 vaccine	NCT02285816
Reovirus	Reolysin	None	Bladder	I	IT	Gemcitabine + Cisplatin	NCT02723838
			Breast	II	IV	Paclitaxel	NCT01656538
			Colorectal	I	IV	FOLFIRI + Bevacizumab	NCT01274624
				II	IV	FOLFOX + Bevacizumab	NCT01622543
			Myeloma	I	IV	Bortezomib + Dexamethasone	NCT02514382
						Lenalidomide or Pomalidomide	NCT03015922
			Pancreas	I	IV	Pembrolizumab + Chemo	NCT02620423
				II	IV	Carboplatin + Paclitaxel	NCT01280058
			Pediatric (brain)	I	IV	GM-CSF	NCT02444546
			Solid tumors	II	IV	Paclitaxel	NCT01199263
Vaccinia	GL-ONC1	*Increased tumor sensitivity to drug and reduced virulence*: TK disruption, hemagglutinin disruption, F14.5L disruption	MPE	I	IPl	–	NCT01766739
			Ovarian	I	IP	–	NCT02759588
			Solid tumors	I	IV	Eculizumab	NCT02714374
	JX-594	*Immune activation and increased tumor sensitivity to drug*: GM-CSF insertion, TK disruption	Breast, sarcoma	I/II	IV	CPA	NCT02630368
			HCC	III	IT	Sorafenib	NCT02562755
			Solid tumors	I	IT	Ipilimumab	NCT02977156
	PROSTVAC	*Tumor antigen to enhance antitumor immune activity*: PSA, LFA-3, ICAM-1, B7.1 additions	Prostate	I/II	SC	Nivolumab and/or Ipilimumab	NCT02933255
				II	SC	–	NCT02326805
						–	NCT02649439
						–	NCT02772562
						Ipilimumab	NCT02506114
						Docetaxel + Prednisone	NCT01145508
						Docetaxel	NCT02649855
						Flutamide	NCT00450463
						–	NCT02153918
						Enzalutamide	NCT01867333
							NCT01875250
				III	SC	GM-CSF	NCT01322490
Vesicular stomatitis	*VSV-IFN*β*-NIS*	*Increased tumor specificity and enhanced sensitivity to radiotherapy*: IFN-β + NIS	Hematologic malignancy	I	IV	–	NCT03017820
			Solid tumors	I	IV	–	NCT02923466

*CPA, cyclophosphamide; IM, intramuscular; IP, intraperitoneal; IPl, intrapleural; IT, intratumoral; IV, intravenous; MPE, malignant pleural effusion; SC, subcutaneous; RT, radiotherapy*.

**Figure 1:**
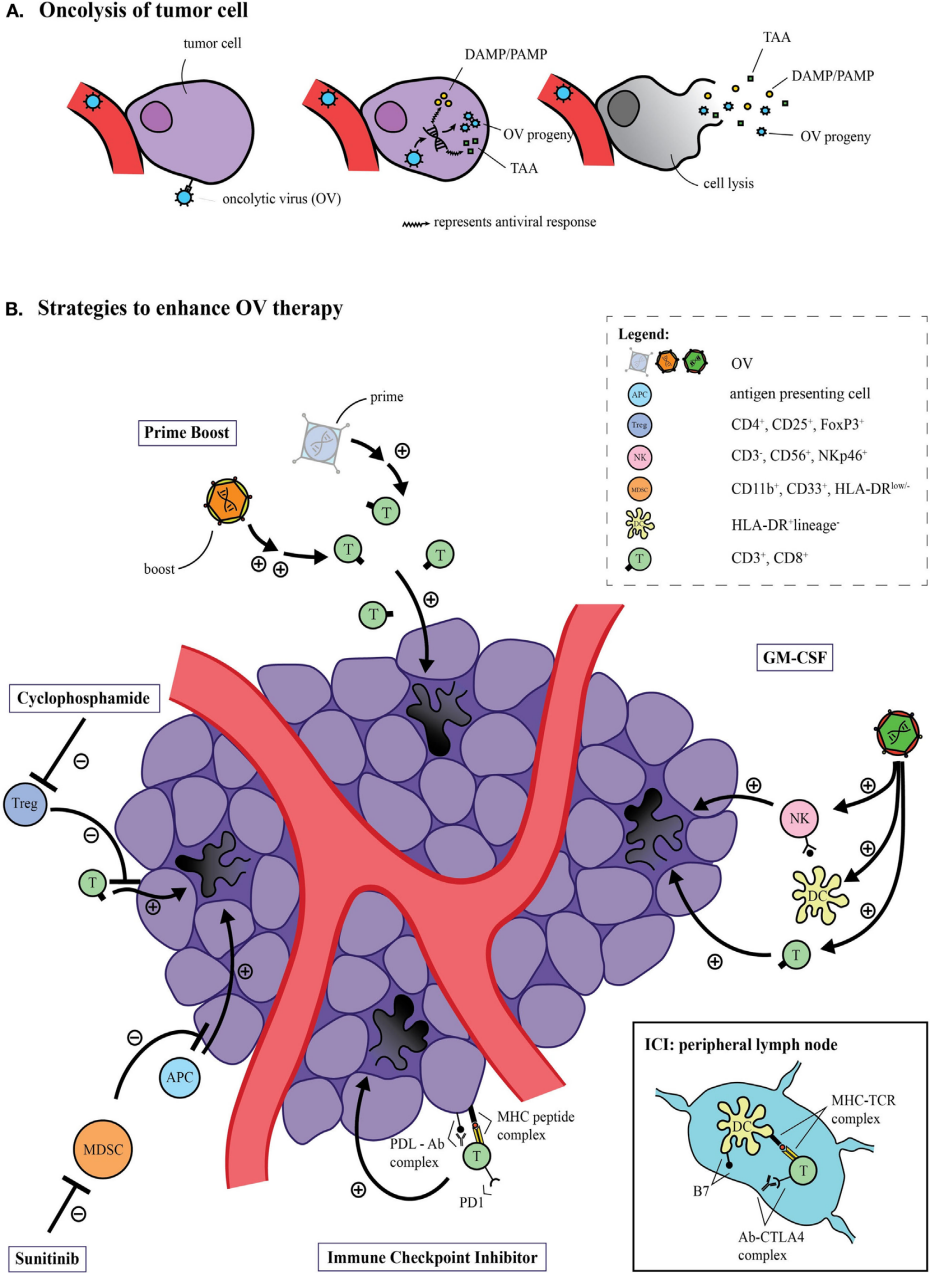
Oncolytic virus (OV)-mediated tumor cell lysis. **(A)** OV can specifically infect cancer cells, and subsequent replication can induce oncolysis. The release of tumor antigens has the potential to activate a systemic antitumor immune response. **(B)** The immune response induced by OV can be improved through several strategies. The prime-boost approach utilizes one priming viral platform carrying tumor-specific antigens, while a second platform—usually an OV—carrying the same antigens boosts the resultant antitumor immune response. The insertion of transgenes, such as GM-CSF, can facilitate antigen presentation on the surface of dendritic cells, and thus augment an antitumor response by recruiting natural killer (NK) cells and inducing tumor-specific cytotoxic T-cells. Immune checkpoint inhibitors can function both at the level of the tumor, targeting the programmed cell death protein 1 (PD-1) axis or peripherally at the level of the lymph nodes by targeting the cytotoxic T lymphocyte-associated antigen 4 (CTLA-4) axis. Both approaches ultimately improve the antitumor response. Immunomodulatory drugs such as sunitinib and cyclophosphamide can augment the antitumor immune response of OV by inhibiting immunosuppressive populations, such as myeloid-derived suppressor cells (MDSCs) and regulatory T cells (Tregs), respectively.

OV therapy is based on the finding that certain viruses selectively replicate within cancer cells. Initially, OV therapy was thought to exert its primary anticancer effect through direct tumor oncolysis (apoptosis/autophagy). However, almost 20 years ago, findings by Mastrangelo and colleagues (5) demonstrated that, in fact, another mechanism may be at play with oncolytic virotherapy. Not only did primary tumors decrease in size when injected with an oncolytic vaccinia virus (VV), but non-injected tumors did as well (5). Their findings suggested that OV have the potential to induce systemic antitumor immunity. It is now commonly accepted that exposure of tumor neoantigens after OV-induced oncolysis (Figure 1A) can activate both the innate and adaptive arms of the host immune system and direct them specifically toward areas of tumor burden. It is currently unclear to what extent each of these mechanisms contributes to the overall success of clinical efficacy in an individual OV.

Interestingly, there has been only modest success in the introduction of OV to the clinical arena as monotherapies (6, 7). The explanation for these modest results is likely multifactorial, including host antiviral mechanisms limiting effective viral dissemination, development of tumor resistance to key oncogenic signaling pathways typically exploited by OV, and a host of immunosuppressive regulatory factors within the tumor microenvironment. Current clinical approaches utilizing OV seek to enhance their efficacy with complimentary immunotherapeutic strategies (Figure 1B).

As the field of OV is in the midst of renewed excitement and optimism, we seek herein to provide an overview of the most frequently utilized immune-based strategies to improve the clinical efficacy of oncolytic virotherapy and review the available evidence for doing so.

### Manipulating OV for Clinical Benefit

#### The Hallmark Transgene: GM-CSF

Early in the process of bringing OV into the clinical setting, it was realized that certain viral candidates could be genetically modified to reduce virulence and/or be armed with therapeutic transgenes to augment oncolytic activity with local gene delivery. Transgenes to enhance therapeutic benefit of OV are quite varied and include inflammatory cytokines, proteases that degrade the tumor microenvironment, antiangiogenic proteins, prodrug-converting enzymes, and proapoptotic genes (8). In general, the trend in the OV field is to enhance candidate viruses in such a way that their ability to induce antitumor immunity is optimized. No transgene has been utilized as frequently or with as much success as GM-CSF.

Ever since the antitumor effects of GM-CSF were first appreciated by Dranoff and colleagues (9), it has held particular interest as a therapeutic adjuvant in immune-based cancer treatments. Based on its effects in cytokine-transduced cancer cell vaccines such as Sipuleucel-T for prostate cancer, it has become an attractive OV therapeutic transgene. By promoting monocyte-to-dendritic cell (DC) differentiation, GM-CSF facilitates antigen presentation on the surface of DCs following viral-induced oncolysis (10). This ultimately leads to a more robust antitumor immune response by recruiting natural killer (NK) cells and inducing tumor-specific cytotoxic T-cells (11).

To date, GM-CSF has been used with success in OV platforms such as HSV (4, 12), VV (13, 14), and adenovirus (AdV) (15, 16). Of these, HSV and VV have arguably served as the most efficacious platforms. A phase III randomized clinical trial comparing HSV-1 with a GM-CSF Transgene Talminogene laherparepvec (T-VEC) vs. GM-CSF alone in advanced melanoma led to the first FDA approval of an OV. Of 436 patients randomized, 295 were in the T-VEC group and 141 in the GM-CSF arm. The objective response rate (ORR) was 26.4% for T-VEC, including 10.8% with a complete clinical response, vs. 5.7% for GM-CSF alone. Despite not quite reaching statistical significance, those in the T-VEC arm achieved an overall survival of 23.3 vs. 18.9 months in the GM-CSF group, thus demonstrating a meaningful trend toward improved survival (4).

The utility and efficacy of T-VEC are currently being explored across a variety of cancer types with phase II clinical trials open in breast (NCT02658812), lymphoma (NCT02978625), and sarcoma (NCT02923778). Additionally, another randomized phase III trial in melanoma is open exploring the value of adding T-VEC to the programmed cell death protein 1 (PD-1) inhibitor, pembrolizumab, for treatment of unresected melanoma (NCT02263508).

Furthermore, an oncolytic VV has been programmed with a GM-CSF insertion (JX-594) and has been the subject of much clinical investigation. Early-phase I/II trials have been completed with JX-594 in colorectal cancer (17), melanoma (18), pediatric malignancy (19), and non-specific solid tumors (14). The greatest clinical promise, however, has been seen with JX-594 in hepatocellular carcinoma (HCC). A phase II dose-finding trial demonstrated significant survival benefit with high doses (14.1 months) compared to low doses (6.7 months) of JX-594 (20). Furthermore, it was found that objective tumor responses were present in both injected- and non-injected tumors, indicating a possible element of systemic antitumor immunity. Studies of this OV in a preclinical setting have demonstrated that tumor oncolysis is mediated by antibodies in a complement-dependent nature (21), likely related to its ability to increase the release of specific tumor neoantigens/epitopes to the systemic circulation. Further exploration of its efficacy in HCC is currently ongoing, with a phase III trial open for recruitment (NCT02562755) with or without with the VEGFR tyrosine kinase inhibitor Sorafenib.

It is important to consider that despite the clinical promise of OV expressing a GM-CSF transgene, the underlying mechanisms mediating antitumor activity are both poorly understood and subject to controversy. There are little data surrounding the specific mechanistic contributions of GM-CSF to the success of the OV previously mentioned. Moreover, despite the recognition that GM-CSF has a certain level of antitumor potency, it is also intricately linked to the modulation (increase) of immunosuppressive myeloid-derived suppressor cells (MDSCs) (22). Specifically, not only has GM-CSF been shown to increase MDSC numbers in transplantable tumor models (23) but it has also been implicated as the main factor driving MDSC generation in these models (24). Thus, further study is needed to determine the best use of GM-CSF with OV in order to maximize its antitumor effects, while minimizing its recruitment and proliferation of immunosuppressive MDSCs.

#### “Boosting” OV Efficacy: The Prime-Boost Strategy

Based on the success of traditional vaccinations to combat virally induced disease, vaccinating patients with tumor antigens has been a therapeutic approach of interest in cancer, although has only demonstrated modest success to date. Eliciting a successful systemic immune response against tumor antigens requires the breaking of tolerance that typically prevents host antitumor immunity. One answer may be to utilize viral delivery platforms. One problem with this approach lies in that the use of viral vectors may induce a competitive immune response against the viral antigens, rather than the tumor antigens of interest (25). A solution is to utilize the emerging heterologous “prime-boost” approach. For example, tumor-specific antigens can be encoded into the backbone of one viral platform to prime the immune system before being introduced to a second viral platform carrying the same antigens that upregulates, or boosts, the resultant antitumor immune response.

Classic viral vaccine vectors are non-replicating and therefore do not have oncolytic properties. However, the prime-boost strategy with non-OV has still seen demonstrable clinical applicability. PROSTVAC, which is utilized in prostate cancer, is the prototypical example. Despite not utilizing an OV platform, ongoing clinical trials of PROSTVAC are highlighted in Table 1, as success of this platform to date demonstrates the power of the prime-boost strategy in viral-based cancer vaccination.

There are two members of the *Rhabdoviridae* family that have been investigated for use as OV, both belonging to the *Vesiculovirus* genus—vesicular stomatitis virus (VSV) and Maraba virus. These enveloped ssRNA viruses were first noted to have oncolytic potential in 2000 when VSV was demonstrated to induce tumor regression in a mouse xenograft model of melanoma (26). VSV is a promising oncolytic agent due to its reasonable safety profile and lack of preexisting neutralizing antibodies in humans—problems that have been encountered with other OV platforms. It has been demonstrated that VSV can be utilized effectively as a cancer vaccine, with increased capacity as part of a heterologous prime-boost strategy (27, 28). In a murine model of melanoma, VSV vaccine not only induced upregulation of tumor-specific immunity but also decreased adaptive antiviral immunity leading to an increase in the overall survival of treated animals (27). Following the early preclinical success of VSV, other mammalian cell-trophic rhabdovirus family members were screened for oncolytic capacity (29). From this study, Maraba virus was identified as having the broadest oncotropism, which could be further enhanced with the induction of two-point mutations (L123W in M and Q242R in G). In a direct comparison to a similarly mutated VSV in a murine model of metastatic colorectal cancer, this Maraba virus (MG1) induced total tumor clearance in 100% of treated animals, as compared to 30% in VSV (29). Later studies specifically investigating a Maraba MG1 expressing a melanoma antigen demonstrated its inability to prime an adaptive immune response but significant capacity as a boosting vector. In a syngeneic murine model of melanoma, utilizing Maraba MG1 had dramatic effects leading to significantly extended median survival and complete remission of 20% of animals treated (30). Preclinical promise has allowed Maraba MG1 to move into early-phase clinical trials, with two currently ongoing (NCT02879760, NCT02285816). Both trials utilize a non-replicating AdV vector for priming with MG1 as the boost. Results are not yet available.

### Synergistic Strategies with OV and Immune-Modulatory Drugs

#### Cyclophosphamide (CPA)

Cyclophosphamide is a commonly used anticancer agent that non-specifically causes DNA alkylation and induces apoptotic cell death. Additionally, CPA can modulate the immune system through its ability to kill proliferating NK cells, T cells, and B cells with relatively low clinical doses (31). Thus, CPA has been investigated for a synergistic effect along with OV and has demonstrated improved tumor destruction in preclinical models of reovirus (RV) (32, 33), VV (34), measles (35), and AdV (36). Specifically, in a murine model of melanoma, preconditioning with CPA led to an increased intratumoral viral level of oncolytic RV and led to enhanced antitumor efficacy (32). Additionally, one study demonstrated that CPA treatment in conjunction with OV therapy leads to control of the host antiviral response, a problem that can dampen effective OV proliferation, especially in viral platforms that are ubiquitous in humans (37). Furthermore, CPA can potentiate OV replication by suppressing local innate immune cells (38) and depleting regulatory T cells (Tregs), thus enhancing antitumor activity of cytotoxic T-cells (11). Recently, a number of early-phase clinical trials investigating OV synergy with CPA have been completed in oncolytic AdV (solid tumors) (15), oncolytic RV (pediatric tumors, solid tumors) (39, 40), and oncolytic Seneca Valley Virus (neuroendocrine tumors) (41). These trials, however, did not examine the role of CPA specifically in advancing the efficacy of the OV platforms. Furthermore, two current early-phase clinical trials utilizing CPA and an AdV platform are being conducted (NCT00634231, NCT02879669) as well as one trial utilizing CPA and an oncolytic VV (NCT02630368). The general landscape of cancer immune therapies, however, is gravitating toward more tumor-specific therapies. As such, other immune-modulatory agents are being explored, and CPA’s role as a synergistic treatment strategy to compliment OV therapy is diminishing.

#### Immune Checkpoint Inhibitors (ICIs)

Immune checkpoint inhibitors function as immune suppression antagonists. Normally crucial for the maintenance of self-tolerance, immune checkpoint proteins can be overexpressed by tumors as a way to evade detection by the host immune system (42). The first immune checkpoint to be targeted for therapeutic benefit was cytotoxic T lymphocyte-associated antigen 4 (CTLA-4), but superior clinical outcomes, broader clinical applications, and more favorable safety profiles have led PD-1 and its cognate ligand (PD-L1) inhibition to be the new vogue. Importantly, PD-1/PD-L1 inhibition can be combined with CTLA-4 antagonists. PD-L1 expression specifically is induced on activated T cells following a stimulatory signal from IFN-γ (43). CTLA-4 acts at the level of the draining lymph node for T cell priming. Conversely, the PD-1/PD-L1 pathway only inhibits activated T cells, which attenuates the potential for loss of self-tolerance. Since many tumors overexpress PD-L1 (44), they can escape recognition by tumor-infiltrating lymphocytes. Inhibiting this pathway effectively “removes the brakes” on the normal immune response. The impressive success of PD-1/PD-L1 inhibition as monotherapy in phase III clinical trials of melanoma (45), non-small cell lung cancer (46), renal cell carcinoma (RCC) (47), and urothelial carcinoma (48) has led to FDA approval for clinical use. One crucial problem with ICI is that despite their profound efficacy in responding patients, the majority of patients are non-responders (49, 50). This can possibly be explained by the lack of active tumor-specific T cells in the tumor microenvironment. As OV therapy can induce antitumor adaptive immunity, it seems as though ICI and OV could be a perfect therapeutic match.

Preclinical success marrying ICI with OV therapy has been encouraging. Specifically, a study conducted by Zamarin and colleagues (51) demonstrated the potential for combining CTLA-4 inhibition with an oncolytic Newcastle disease virus in a murine model of melanoma. They found that OV therapy alone triggered a systemic antitumor immune response, but accumulated T cells overexpressed CTLA-4, leading to an immunosuppressive tumor microenvironment and diminished treatment efficacy. Adding in CTLA-4 inhibition, however, improved the antitumor response, leading to increased long-term survival of dually treated animals. This response was dependent on NK cells, CD8^+^ T cells, and type I interferon (51). Although still ongoing, one clinical trial (NCT01740297) utilizing T-VEC and CTLA-4 blockade has promising interim results; ORR has been found in 41% of treated patients and complete responses in 24%. Given that T-VEC monotherapy has a reported ORR of 26% and a complete response rate of 10.8% (4), the combination therapy with CTLA-4 blockade seems to be an improvement. Additionally, a preclinical study in a murine model of melanoma utilizing an oncolytic RV in combination with PD-1 inhibition demonstrated promising results (52). This group found that combination treatment significantly enhances survival compared to either monotherapy. The enhanced survival was tied to increased activity of NK cells, reduced Tregs, and increased CD8^+^ antitumor responses (52). Between PD-1 inhibitors nivolumab and pembrolizumab, PD-Ll inhibitor durvalumab, and CTLA-4 inhibitor ipilimumab, there are currently 19 clinical trials ongoing that combine ICI and OV (Table 1). Results from these trials are eagerly anticipated in order to assess the value of combining these two immune-based treatment modalities.

#### Sunitinib

Sunitinib is a multi-tyrosine kinase inhibitor (VEGFR, PDGFR, c-kit, flt3, RET, CSF-1R) that has FDA approval for use in RCC and gastrointestinal stromal tumors. Its primary antitumor effect is through inhibition of VEGFR, leading to a reduced capacity for tumor angiogenesis (53). It is also now understood that sunitinib also has a role in indirectly inhibiting tumor growth through the promotion of antitumor immune responses (54–56). For example, immunosuppressive immune cell populations such as Tregs and MDSC are decreased with sunitinib treatment (54, 55). Its role as an immunotherapeutic adjuvant makes it a suitable candidate for combination with OV. Interestingly, it has been demonstrated that sunitinib can lead to the enhancement of viral replication through targeting innate immune pathways of viral resistance such as double-stranded RNA protein Kinase R (PKR) and RNase (57). The timing of sunitinib administration seems to be of importance, as administering it prior to and during oncolytic RV therapy allowed for the preconditioning of the tumor microenvironment to facilitate a maximal OV-induced antitumor response (58). Although no clinical trials have been initiated utilizing sunitinib and OV, one preclinical study seems to suggest potential for this combination in the treatment of RCC. Sunitinib and an oncolytic RV were found to significantly decrease tumor burden and significantly increase lifespan in a murine model of RCC (59). This therapeutic effect could be explained by their finding that this treatment combination increased the presence of tumor-specific CD8^+^ T cells and decreased accumulation of both MDSCs and Tregs. Additionally, dually treated mice had protective immunity upon tumor rechallenge. In the same study, Lawson and colleagues (59) also demonstrated similar results in a murine model of squamous cell lung carcinoma, thus highlighting the possible broad application of this treatment strategy. Furthermore, sunitinib combination with an oncolytic VSV led to the elimination of prostate, breast, and kidney malignant tumors in mice (60). Additionally, the antiangiogenic effects of sunitinib can be augmented by the utilization of an oncolytic VV, leading to reduction of tumor growth in murine models of cancer (61). Hopefully, the preclinical success of sunitinib and OV will be replicated in clinical trials once they are initiated.

## Other Strategies to Enhance OV

Although the focus of this review has been necessarily limited to a handful of combinatorial immunotherapeutic strategies to enhance OV therapy, there are a number of other exciting approaches under preclinical investigation. For example, the combination of adoptive T cell therapy with OV has shown preclinical promise and efforts are underway to bring this strategy to clinical investigation (62, 63). Additionally, a number of different OV platforms are being utilized in combination with inhibitors of histone deacetylases (HDACIs) [reviewed in Ref. (64)]. Although the mechanisms underpinning their tumor tropism are not fully understood (65), HDACIs led to immunogenic cell death of cancer cells thus potentially enhancing antitumor immune responses in synergy with OV (66, 67).

Finally, a transgene-modified oncolytic AdV, NG-348 (PsiOxus Therapeutics), has been recently designed in hopes that it will drive T-cell immune responses within the tumor microenvironment independent of tumor-specific antigens. When two transgenes, a membrane anchored full-length human CD80 and a membrane anchored antibody fragment for the T-cell receptor, are expressed together on the surface of NG-348-infected tumor cells they provide both the T-cell receptor and costimulatory signal required to activate tumor-infiltrating T-cells (68). This strategy mimics that of CAR-T therapies but does not require autologous cell processing or tumor-specific antigens. Furthermore, since the expression of the encoded transgenes is encoded by the endogenous viral major late promoter, their expression is limited to the surface of cells permissive to viral infection—i.e. tumor cells. It is hoped that preclinical testing of NG-348 will ultimately support clinical application.

## Concluding Remarks

Oncolytic viruses represent a promising immunotherapeutic approach to the treatment of cancer. Although clinical trials have demonstrated that their use as a monotherapy is likely insufficient for meaningful efficacy in the clinical arena, it has become clear that the ability for OV to induce a systemic antitumor immune response is intricately linked to their potential for success. Therefore, combining OV with other immunotherapies seems to represent the approach with the most promise. As numerous clinical trials are underway across multiple OV platforms utilizing different immunotherapies for treatment synergy, time will ultimately unveil the potential for OV as a future standard treatment option for our patients with cancer.

## Author Contributions

DM is the primary author of this manuscript. AW designed and produced the included figure. All authors assisted in the conception of this review, acquisition of relevant literature, and editing the manuscript. All authors gave approval of the final version to be published.

## Conflict of Interest Statement

The authors declare that the research was conducted in the absence of any commercial or financial relationships that could be construed as a potential conflict of interest.

## References

[1] Siegel RL Miller KD Jemal A. Cancer statistics, 2016. CA Cancer J Clin (2016) 66:7–30. 10.3322/caac.21332 26742998

[2] Subarsky P Hill RP. The hypoxic tumour microenvironment and metastatic progression. Clin Exp Metastasis (2003) 20:237–50. 10.1023/A:1022939318102 12741682

[3] Dock G The influence of complicating diseases upon leukaemia. Am J Med Sci (1904) 127:563–92. 10.1097/00000441-190412740-00001

[4] Andtbacka RHI Kaufman HL Collichio F Amatruda T Senzer N Chesney J Talimogene laherparepvec improves durable response rate in patients with advanced melanoma. J Clin Oncol (2015) 33:2780–8. 10.1200/JCO.2014.58.3377 26014293

[5] Mastrangelo MJ Maguire HC Eisenlohr LC Laughlin CE Monken CE McCue PA Intratumoral recombinant GM-CSF-encoding virus as gene therapy in patients with cutaneous melanoma. Cancer Gene Ther (1999) 6:409–22. 10.1038/sj.cgt.7700066 10505851

[6] Patel MR Kratzke RA. Oncolytic virus therapy for cancer: the first wave of translational clinical trials. Transl Res (2013) 161:355–64. 10.1016/j.trsl.2012.12.010 23313629

[7] Pol J Buqué A Aranda F Bloy N Cremer I Eggermont A Trial watch-oncolytic viruses and cancer therapy. Oncoimmunology (2016) 5:e1117740 10.1080/2162402X.2015.1117740 27057469 PMC4801444

[8] Sampath P Thorne SH Arming viruses in multi-mechanistic oncolytic viral therapy: current research and future developments, with emphasis on poxviruses. Oncolytic Virother (2014) 3:1–9. 10.2147/OV.S36703 27512659 PMC4918358

[9] Dranoff G Jaffee E Lazenby A Golumbek P Levitsky H Brose K Vaccination with irradiated tumor cells engineered to secrete murine granulocyte-macrophage colony-stimulating factor stimulates potent, specific, and long-lasting anti-tumor immunity. Proc Natl Acad Sci U S A (1993) 90:3539–43. 10.1073/pnas.90.8.3539 8097319 PMC46336

[10] Lichty BD Breitbach CJ Stojdl DF Bell JC. Going viral with cancer immunotherapy. Nat Rev Cancer (2014) 14:559–67. 10.1038/nrc3770 24990523

[11] Bartlett DL Liu Z Sathaiah M Ravindranathan R Guo Z He Y Oncolytic viruses as therapeutic cancer vaccines. Mol Cancer (2013) 12(1):103 10.1186/1476-4598-12-103 24020520 PMC3847443

[12] Liu BL Robinson M Han Z-Q Branston RH English C Reay P ICP34.5 deleted herpes simplex virus with enhanced oncolytic, immune stimulating, and anti-tumour properties. Gene Ther (2003) 10:292–303. 10.1038/sj.gt.3301885 12595888

[13] Park B-H Hwang T Liu T-C Sze DY Kim J-S Kwon H-C Use of a targeted oncolytic poxvirus, JX-594, in patients with refractory primary or metastatic liver cancer: a phase I trial. Lancet Oncol (2008) 9:533–42. 10.1016/S1470-2045(08)70107-4 18495536

[14] Breitbach CJ Burke J Jonker D Stephenson J Haas AR Chow LQM Intravenous delivery of a multi-mechanistic cancer-targeted oncolytic poxvirus in humans. Nature (2011) 477:99–102. 10.1038/nature10358 21886163

[15] Ranki T Pesonen S Hemminki A Partanen K Kairemo K Alanko T Phase I study with ONCOS-102 for the treatment of solid tumors – an evaluation of clinical response and exploratory analyses of immune markers. J Immunother Cancer (2016) 4:17 10.1186/s40425-016-0121-5 26981247 PMC4791966

[16] Cerullo V Pesonen S Diaconu I Escutenaire S Arstila PT Ugolini M Oncolytic adenovirus coding for granulocyte macrophage colony-stimulating factor induces antitumoral immunity in cancer patients. Cancer Res (2010) 70:4297–309. 10.1158/0008-5472.CAN-09-3567 20484030

[17] Park SH Breitbach CJ Lee J Park JO Lim HY Kang WK Phase 1b trial of biweekly intravenous Pexa-Vec (JX-594), an oncolytic and immunotherapeutic vaccinia virus in colorectal cancer. Mol Ther (2015) 23:1532–40. 10.1038/mt.2015.109 26073886 PMC4817877

[18] Hwang T-H Moon A Burke J Ribas A Stephenson J Breitbach CJ A mechanistic proof-of-concept clinical trial with JX-594, a targeted multi-mechanistic oncolytic poxvirus, in patients with metastatic melanoma. Mol Ther (2011) 19:1913–22. 10.1038/mt.2011.132 21772252 PMC3188739

[19] Cripe TP Ngo MC Geller JI Louis CU Currier MA Racadio JM Phase 1 study of intratumoral Pexa-Vec (JX-594), an oncolytic and immunotherapeutic vaccinia virus, in pediatric cancer patients. Mol Ther (2015) 23:602–8. 10.1038/mt.2014.243 25531693 PMC4351466

[20] Heo J Reid T Ruo L Breitbach CJ Rose S Bloomston M Randomized dose-finding clinical trial of oncolytic immunotherapeutic vaccinia JX-594 in liver cancer. Nat Med (2013) 19:329–36. 10.1038/nm.3089 23396206 PMC4268543

[21] Kim MK Breitbach CJ Moon A Heo J Lee YK Cho M Oncolytic and immunotherapeutic vaccinia induces antibody-mediated complement-dependent cancer cell lysis in humans. Sci Transl Med (2013) 5:185ra63. 10.1126/scitranslmed.3005361 23677592

[22] Fernández A Oliver L Alvarez R Fernández LE Lee KP Mesa C Adjuvants and myeloid-derived suppressor cells: enemies or allies in therapeutic cancer vaccination. Hum Vaccin Immunother (2014) 10:3251–60. 10.4161/hv.29847 25483674 PMC4514045

[23] Serafini P Carbley R Noonan KA Tan G Bronte V Borrello I. High-dose granulocyte-macrophage colony-stimulating factor-producing vaccines impair the immune response through the recruitment of myeloid suppressor cells. Cancer Res (2004) 64:6337–43. 10.1158/0008-5472.CAN-04-0757 15342423

[24] Bayne LJ Beatty GL Jhala N Clark CE Rhim AD Stanger BZ Tumor-derived granulocyte-macrophage colony-stimulating factor regulates myeloid inflammation and T cell immunity in pancreatic cancer. Cancer Cell (2012) 21:822–35. 10.1016/j.ccr.2012.04.025 22698406 PMC3575028

[25] Harrop R John J Carroll MW. Recombinant viral vectors: cancer vaccines. Adv Drug Deliv Rev (2006) 58:931–47. 10.1016/j.addr.2006.05.005 17030074

[26] Stojdl DF Lichty B Knowles S Marius R Atkins H Sonenberg N Exploiting tumor-specific defects in the interferon pathway with a previously unknown oncolytic virus. Nat Med (2000) 6:821–5. 10.1038/77558 10888934

[27] Bridle BW Boudreau JE Lichty BD Brunellière J Stephenson K Koshy S Vesicular stomatitis virus as a novel cancer vaccine vector to prime antitumor immunity amenable to rapid boosting with adenovirus. Mol Ther (2009) 17:1814–21. 10.1038/mt.2009.154 19603003 PMC2835010

[28] Pulido J Kottke T Thompson J Galivo F Wongthida P Diaz RM Using virally expressed melanoma cDNA libraries to identify tumor-associated antigens that cure melanoma. Nat Biotechnol (2012) 30:337–43. 10.1038/nbt.2157 22426030 PMC3891505

[29] Brun J McManus D Lefebvre C Hu K Falls T Atkins H Identification of genetically modified Maraba virus as an oncolytic rhabdovirus. Mol Ther (2010) 18:1440–9. 10.1038/mt.2010.103 20551913 PMC2927055

[30] Pol JG Zhang L Bridle BW Stephenson KB Rességuier J Hanson S Maraba virus as a potent oncolytic vaccine vector. Mol Ther (2014) 22:420–9. 10.1038/mt.2013.249 24322333 PMC3916044

[31] Sistigu A Viaud S Chaput N Bracci L Proietti E Zitvogel L. Immunomodulatory effects of cyclophosphamide and implementations for vaccine design. Semin Immunopathol (2011) 33:369–83. 10.1007/s00281-011-0245-0 21611872

[32] Kottke T Thompson J Diaz RM Pulido J Willmon C Coffey M Improved systemic delivery of oncolytic reovirus to established tumors using preconditioning with cyclophosphamide-mediated Treg modulation and interleukin-2. Clin Cancer Res (2009) 15:561–9. 10.1158/1078-0432.CCR-08-1688 19147761 PMC3046733

[33] Qiao J Wang H Kottke T White C Twigger K Diaz RM Cyclophosphamide facilitates antitumor efficacy against subcutaneous tumors following intravenous delivery of reovirus. Clin Cancer Res (2008) 14:259–69. 10.1158/1078-0432.CCR-07-1510 18172278 PMC3046731

[34] Lun XQ Jang JH Tang N Deng H Head R Bell JC Efficacy of systemically administered oncolytic vaccinia virotherapy for malignant gliomas is enhanced by combination therapy with rapamycin or cyclophosphamide. Clin Cancer Res (2009) 15:2777–88. 10.1158/1078-0432.CCR-08-2342 19351762

[35] Ungerechts G Frenzke ME Yaiw K-C Miest T Johnston PB Cattaneo R. Mantle cell lymphoma salvage regimen: synergy between a reprogrammed oncolytic virus and two chemotherapeutics. Gene Ther (2010) 17:1506–16. 10.1038/gt.2010.103 20686506 PMC2976793

[36] Cerullo V Diaconu I Kangasniemi L Rajecki M Escutenaire S Koski A Immunological effects of low-dose cyclophosphamide in cancer patients treated with oncolytic adenovirus. Mol Ther (2011) 19:1737–46. 10.1038/mt.2011.113 21673660 PMC3182348

[37] Peng K-W Myers R Greenslade A Mader E Greiner S Federspiel MJ Using clinically approved cyclophosphamide regimens to control the humoral immune response to oncolytic viruses. Gene Ther (2013) 20:255–61. 10.1038/gt.2012.31 22476202 PMC3806053

[38] Fulci G Breymann L Gianni D Kurozomi K Rhee SS Yu J Cyclophosphamide enhances glioma virotherapy by inhibiting innate immune responses. Proc Natl Acad Sci U S A (2006) 103:12873–8. 10.1073/pnas.0605496103 16908838 PMC1568940

[39] Kolb EA Sampson V Stabley D Walter A Sol-Church K Cripe T A phase I trial and viral clearance study of reovirus (Reolysin) in children with relapsed or refractory extra-cranial solid tumors: a Children’s Oncology Group phase I consortium report. Pediatr Blood Cancer (2015) 62:751–8. 10.1002/pbc.25464 25728527 PMC4376570

[40] Roulstone V Khan K Pandha HS Rudman S Coffey M Gill GM Phase I trial of cyclophosphamide as an immune modulator for optimizing oncolytic reovirus delivery to solid tumors. Clin Cancer Res (2015) 21:1305–12. 10.1158/1078-0432.CCR-14-1770 25424857 PMC4821068

[41] Rudin CM Poirier JT Senzer NN Stephenson J Loesch D Burroughs KD Phase I clinical study of Seneca Valley Virus (SVV-001), a replication-competent picornavirus, in advanced solid tumors with neuroendocrine features. Clin Cancer Res (2011) 17:888–95. 10.1158/1078-0432.CCR-10-1706 21304001 PMC5317273

[42] Pardoll DM. The blockade of immune checkpoints in cancer immunotherapy. Nat Rev Cancer (2012) 12:252–64. 10.1038/nrc3239 22437870 PMC4856023

[43] Dong H Strome SE Salomao DR Tamura H Hirano F Flies DB Tumor-associated B7-H1 promotes T-cell apoptosis: a potential mechanism of immune evasion. Nat Med (2002) 8:793–800. 10.1038/nm730 12091876

[44] Teng MWL Ngiow SF Ribas A Smyth MJ. Classifying cancers based on T-cell infiltration and PD-L1. Cancer Res (2015) 75:2139–45. 10.1158/0008-5472.CAN-15-0255 25977340 PMC4452411

[45] Robert C Long GV Brady B Dutriaux C Maio M Mortier L Nivolumab in previously untreated melanoma without BRAF mutation. N Engl J Med (2015) 372:320–30. 10.1056/NEJMoa1412082 25399552

[46] Brahmer J Reckamp KL Baas P Crinò L Eberhardt WEE Poddubskaya E Nivolumab versus docetaxel in advanced squamous-cell non-small-cell lung cancer. N Engl J Med (2015) 373:123–35. 10.1056/NEJMoa1504627 26028407 PMC4681400

[47] Motzer RJ Escudier B McDermott DF George S Hammers HJ Srinivas S Nivolumab versus everolimus in advanced renal-cell carcinoma. N Engl J Med (2015) 373:1803–13. 10.1056/NEJMoa1510665 26406148 PMC5719487

[48] Rosenberg JE Hoffman-Censits J Powles T van der Heijden MS Balar AV Necchi A Atezolizumab in patients with locally advanced and metastatic urothelial carcinoma who have progressed following treatment with platinum-based chemotherapy: a single-arm, multicentre, phase 2 trial. Lancet (2016) 387:1909–20. 10.1016/S0140-6736(16)00561-4 26952546 PMC5480242

[49] Topalian SL Hodi FS Brahmer JR Gettinger SN Smith DC McDermott DF Safety, activity, and immune correlates of anti-PD-1 antibody in cancer. N Engl J Med (2012) 366:2443–54. 10.1056/NEJMoa1200690 22658127 PMC3544539

[50] Wolchok JD Kluger H Callahan MK Postow MA Rizvi NA Lesokhin AM Nivolumab plus ipilimumab in advanced melanoma. N Engl J Med (2013) 369:122–33. 10.1056/NEJMoa1302369 23724867 PMC5698004

[51] Zamarin D Holmgaard RB Subudhi SK Park JS Mansour M Palese P Localized oncolytic virotherapy overcomes systemic tumor resistance to immune checkpoint blockade immunotherapy. Sci Transl Med (2014) 6:226ra32. 10.1126/scitranslmed.3008095 24598590 PMC4106918

[52] Rajani K Parrish C Kottke T Thompson J Zaidi S Ilett L Combination therapy with reovirus and anti-PD-1 blockade controls tumor growth through innate and adaptive immune responses. Mol Ther (2016) 24:166–74. 10.1038/mt.2015.156 26310630 PMC4754544

[53] Mendel DB Laird AD Xin X Louie SG Christensen JG Li G In vivo antitumor activity of SU11248, a novel tyrosine kinase inhibitor targeting vascular endothelial growth factor and platelet-derived growth factor receptors: determination of a pharmacokinetic/pharmacodynamic relationship. Clin Cancer Res (2003) 9:327–37. 12538485

[54] Ko JS Zea AH Rini BI Ireland JL Elson P Cohen P Sunitinib mediates reversal of myeloid-derived suppressor cell accumulation in renal cell carcinoma patients. Clin Cancer Res (2009) 15:2148–57. 10.1158/1078-0432.CCR-08-1332 19276286

[55] Finke JH Rini B Ireland J Rayman P Richmond A Golshayan A Sunitinib reverses type-1 immune suppression and decreases T-regulatory cells in renal cell carcinoma patients. Clin Cancer Res (2008) 14:6674–82. 10.1158/1078-0432.CCR-07-5212 18927310

[56] Ozao-Choy J Ma G Kao J Wang GX Meseck M Sung M The novel role of tyrosine kinase inhibitor in the reversal of immune suppression and modulation of tumor microenvironment for immune-based cancer therapies. Cancer Res (2009) 69:2514–22. 10.1158/0008-5472.CAN-08-4709 19276342 PMC4370269

[57] Jha BK Polyakova I Kessler P Dong B Dickerman B Sen GC Inhibition of RNase L and RNA-dependent protein kinase (PKR) by sunitinib impairs antiviral innate immunity. J Biol Chem (2011) 286:26319–26. 10.1074/jbc.M111.253443 21636578 PMC3143594

[58] Farsaci B Higgins JP Hodge JW. Consequence of dose scheduling of sunitinib on host immune response elements and vaccine combination therapy. Int J Cancer (2012) 130:1948–59. 10.1002/ijc.26219 21633954 PMC3232304

[59] Lawson KA Mostafa AA Shi ZQ Spurrell J Chen W Kawakami J Repurposing sunitinib with oncolytic reovirus as a novel immunotherapeutic strategy for renal cell carcinoma. Clin Cancer Res (2016) 22:5839–50. 10.1158/1078-0432.CCR-16-0143 27220962

[60] Jha BK Dong B Nguyen CT Polyakova I Silverman RH. Suppression of antiviral innate immunity by sunitinib enhances oncolytic virotherapy. Mol Ther (2013) 21:1749–57. 10.1038/mt.2013.112 23732991 PMC3776628

[61] Hou W Chen H Rojas J Sampath P Thorne SH. Oncolytic vaccinia virus demonstrates antiangiogenic effects mediated by targeting of VEGF. Int J Cancer (2014) 135:1238–46. 10.1002/ijc.28747 24474587 PMC4061259

[62] Shim KG Zaidi S Thompson J Kottke T Evgin L Rajani KR Inhibitory receptors induced by VSV viroimmunotherapy are not necessarily targets for improving treatment efficacy. Mol Ther (2017) 25:962–75. 10.1016/j.ymthe.2017.01.023 28237836 PMC5383647

[63] Rommelfanger DM Wongthida P Diaz RM Kaluza KM Thompson JM Kottke TJ Systemic combination virotherapy for melanoma with tumor antigen-expressing vesicular stomatitis virus and adoptive T-cell transfer. Cancer Res (2012) 72:4753–64. 10.1158/0008-5472.CAN-12-0600 22836753 PMC3893932

[64] Marchini A Scott EM Rommelaere J. Overcoming barriers in oncolytic virotherapy with HDAC inhibitors and immune checkpoint blockade. Viruses (2016) 8:9. 10.3390/v8010009 26751469 PMC4728569

[65] Falkenberg KJ Johnstone RW Histone deacetylases and their inhibitors in cancer, neurological diseases and immune disorders. Nat Rev Drug Discov (2014) 13:673–91. 10.1038/nrd4360 25131830

[66] Christiansen AJ West A Banks K-M Haynes NM Teng MW Smyth MJ Eradication of solid tumors using histone deacetylase inhibitors combined with immune-stimulating antibodies. Proc Natl Acad Sci U S A (2011) 108:4141–6. 10.1073/pnas.1011037108 21368108 PMC3054015

[67] Bridle BW Chen L Lemay CG Diallo J-S Pol J Nguyen A HDAC inhibition suppresses primary immune responses, enhances secondary immune responses, and abrogates autoimmunity during tumor immunotherapy. Mol Ther (2013) 21:887–94. 10.1038/mt.2012.265 23295947 PMC3616544

[68] Champion BR Rasiah N Illingworth S Besneux M Lear R Plumb D Developing tumor-localized, combination immunotherapies. Cancer Res (2016) 76 10.1158/1538-7445.AM2016-4875

